# A Collection of Curiosities

**Published:** 1884-11

**Authors:** 


### A COLLECTION OF CURIOSITIES.

The skill of the best diagnostician is, without question, a product of profound study, wide experience, and mastery of many of the innumerable problems of science. This, however, is not all that he requires. There is a chamber of curiosities which he should at times visit, in order to be periodically reminded that even the expert must possess a mental quality which is, perhaps, well described in our language by the familiar phrase, "common sense."

The collection of bric-a-brac in this apartment includes things new and old. Needless to describe, standing just within its portal, the wax-figure of the doctor, in his rusty garments of antiquity, who first mistook a uterus containing a live fœtus for a malignant growth. Only a little less known is his vis-a-vis, the effigy of the famous imposter who, by simulating epilepsy, deceived half the profession in England!

The miscellaneous assortment of articles covering the long tables beyond are not the least valuable of the singular contents of this chamber. They have been responsible for obstinate disturbances and diseases of almost every system of the human body, full of mystery till their agency was explained either by accident or skill; and the professional reputations they have wrecked, no man can number. Here are peas, beans, and other small bodies from the vegetable kingdom; pieces of shells, and fragments of insects; pens, pencils and umbrella-ribs; parts of domestic utensils; and even candles and parts of candlesticks; with who can say what else beside! These have been thrust into the ears, noses, and flesh of innumerable men, women and children; stuffed under their eye-lids; rammed into vagina and uterus; insinuated into urethra and bladder; pushed into the rectum and colon; and even lodged in the umbilicus! Each can tell a sad tale of misguided humanity; each can teach a true lesson to the diagnostician. Here are the round dozen of small files taken from the stomach of a madman in Portugal; here is an entire beer-bottle removed from the bowel of a man in Massachusetts; here is the famous Hodge pessary turned out from the bladder of a woman in Illinois! Here, also, are curious concretions of animal matters which have occasioned no little suffering to the unfortunate people who secreted them, and have tripped up too many a good doctor who was summoned to cure their mischief. Here are scybala, scarcely less solid and bulky than cannon shot; caseous rings cast about the glans penis, as firm as the rind of an ancient cheese; pellets of wax like bullets of lead!

The collection representing the animal kingdom is not without its objects of interest. Here are the larvæ of insects deposited beneath the skin; lice, whose incursions upon the human body have been responsible for many a "humor of the blood"—(poor abused human blood!)—fist-sized masses of inwoven lumbricoids that could deceive the very elect! Ah, what volumes would contain the learned opinions written on the diseases caused by these and their like, the only fault of all which was that neither writers nor readers ever guessed the real truth!

With every year this singular collection grows apace. With every venture of man's ingenuity to make life better worth living, there seems to spring a new source of disease, and hard by it yawns a new pit-fall for the diagnostician. Yonder, for example, are some rather new-looking retorts, containing gaseous emanations from sewers, that have been responsible for ever so many cases of "neurasthenia!" Who was it that lately told the secret that the curious Brazilian disease of the little toe, known there as "ainhum," was produced by tying a string about the diseased organ, under the influence of superstitions prevalent among the negroes of that country?

At the last meeting of the American Dermatological Association, two new contributions were made to this collection of curiosities. One was a ball of blue worsted, employed, innocently enough, by an old lady in knitting stockings, but yet two physicians thought she was suffering from a rare case of blue sweating under the eye-lids, till it was shown that her soiled fingers, applied to that part when irritated, had conveyed the coloring material to it from the worsted. Another was a red-bordered blanket, the edge of which, exposed beyond the sheets of the bed-clothing, had become responsible for the "syphilis" of the unfortunate youth who had slept in its folds and thereby received its aniline stain beneath his chin.

The very latest addition to the museum is contributed by Boston. At first sight one would assuredly not regard it as an object of suspicion. It is only a partially digested oyster. Yet this depraved bivalve lay in wait, like a wolf, for a wandering sheep of a diagnosis. Having obtained access to the stomach of a patient, it refused there to accomplish its destiny of digestion, and had itself thence incontinently regurgitated. On its travels to the upper world again, it found temporary lodging in a convenient tonsil at hand, that asked no questions and demanded no rent. When did a scurvy knave of the world ever fail to find an accomplice in his villainy! Being thus safely lodged, our errant oyster had himself arrested by two pathologists for a neoplasm, a malignant outgrowth from his tonsillar environment; but, his real character having been discovered, his carcase serves, as should those of all convicted rogues, to point an old, old moral, and to adorn a tale that can scarce be too often told.

